# Use of short-course, high-dose clofazimine for type 1 lepra reaction in an HIV-positive patient with leprosy: A case report

**DOI:** 10.1016/j.jdcr.2025.07.008

**Published:** 2025-07-28

**Authors:** Glen Aldrix R. Anarna, Claudine Yap-Silva

**Affiliations:** Department of Dermatology, University of the Philippines – Philippine General Hospital, Manila, Philippines

**Keywords:** high-dose clofazimine, HIV, leprosy, leprosy reactions, type 1 lepra reaction

## Introduction

Leprosy, or Hansen’s disease, is a chronic infectious disease caused by *Mycobacterium leprae* primarily on the skin and the peripheral nerves. It is considered as one of the neglected tropical diseases.^1^ In 2019, the Philippines reported one of the highest number of new cases of leprosy in the World Health Organization Western Pacific Region.^2^ While multidrug therapy (MDT) has reduced reported cases, the disease burden is still high, emphasizing the need for early diagnosis and treatment to prevent lifelong disabilities.^3^ These disabilities, however, do not occur only due to leprosy itself but also in patients who develop severe leprosy reactions during their course of treatment.

Lepra reactions in leprosy are classified into Type 1 (reversal reactions) and Type 2 (erythema nodosum leprosum). Type 1 lepra reactions usually occur in borderline leprosy indicating a change cell-mediated immunity, presenting with edema, erythema of the skin lesions, and neuritis. Treatment involves systemic corticosteroids, gradually tapered down until improvement occurs.^4^ Type 2 lepra reactions, commonly seen in borderline-lepromatous and lepromatous leprosy patients, arise from a systemic inflammatory response due to extravascular immune complex deposition, presenting as new-onset erythematous painful nodules occurring in the trunk and extremities, fever, and uveitis, among others.^5^ Thalidomide is the preferred treatment, although systemic corticosteroids and clofazimine can also be used.^4^

Leprosy in people living with HIV (PLHIV) presents similarly when compared to the general population.^6^ However, management in lepra reactions in PLHIV patients can be challenging due to the immunosuppressive nature of first-line treatments. Caution is necessary with chronic steroid use in PLHIV patients to avoid opportunistic infections, and thus non-immunosuppressive alternatives should be considered.

## Case report

A 40-year-old male presented with a year-long history of multiple, erythematous papules and plaques, initially on both arms and later became generalized, with occasional febrile episodes and arthralgia. Skin punch biopsy of an erythematous plaque revealed dense nodular infiltrates of epithelioid and foamy histiocytes with globi formation and perineural and peri-adnexal infiltrates consistent with lepromatous leprosy. The Fite-Faraco stain was positive for acid-fast bacilli. He was referred to our institution for initiation of MDT for leprosy. He was diagnosed with HIV 3 months prior maintained on lamivudine-tenofovir disoproxil fumarate-dolutegravir.

**Fig 1:**
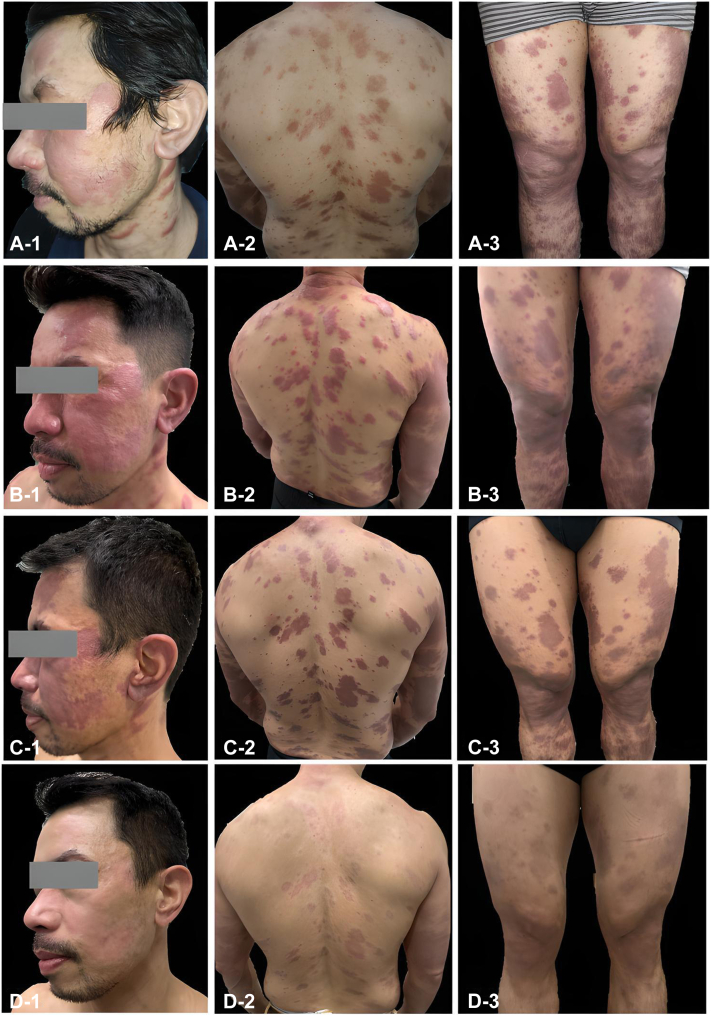
**A-1-A-3,** Skin lesions prior to MDT treatment; (**B-1-B-3**): skin lesions 12 months post-MDT. There is elevation and more erythema of the previously affected areas; (**C-1-C-3**): skin lesions after short-course high-dose clofazimine and extended MDT. There is flattening of the edematous plaques turning into dark brown patches; (**D-1-D-3):** skin lesion 1 year post-treatment. Lesions are less erythematous and less pigmented. *MDT*, Multidrug therapy.

Dermatologic examination showed generalized erythematous papules and plaques with scales and diminished hairs (Fig 1, *A-1-A-3*), without enlarged or tender nerves palpated. Motor and sensory testing was unremarkable. He was initiated on MDT for leprosy, which included rifampicin 600 mg/month, clofazimine 300 mg/month and then 50 mg/day, and dapsone 100 mg/day.

Fifteen days after MDT initiation, he experienced increased in erythema and thickness of skin lesions, along with bipedal edema and left inguinal lymphadenopathy, leading to diagnosis of Type 1 lepra reaction. He was started on prednisone 40 mg daily (0.80 mkd) for 2 weeks. While on prednisone, skin lesions became less elevated, and thus dose was tapered every 2 weeks.

**Fig 2:**
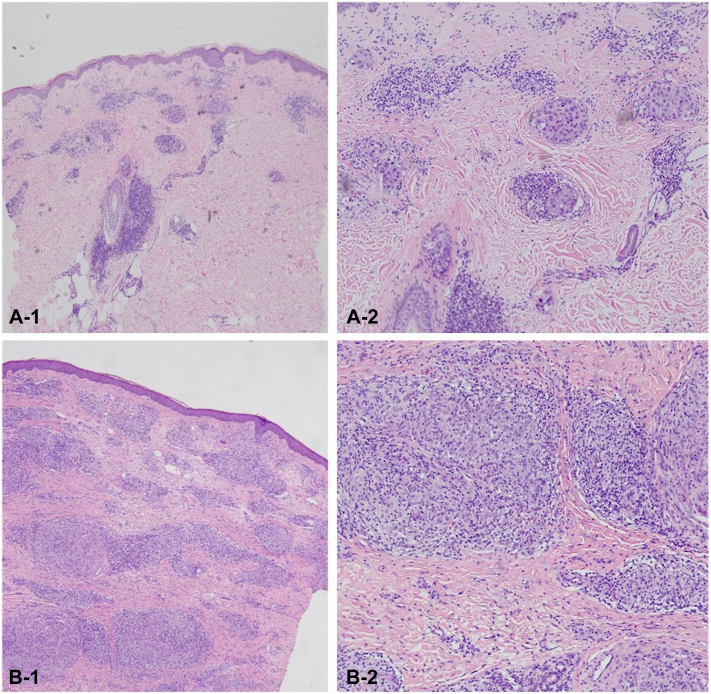
**A-1-A-2,** Histopathologic findings prior to MDT treatment (2022). Note the presence of nodular perivascular, periadnexal, and interstitial infiltrates composed of foamy histiocytes consistent with a lepromatous leprosy; (**B-1-B-2**): histopathologic findings post 12 months of MDT. There is an interval increase in infiltrates, but these are more epithelioid as compared to previous biopsy done. *MDT*, Multidrug therapy.

The lesions were controlled on prednisone 10 mg daily (0.20 mkd) for 10 months. In the 11th month of MDT, repeat CD4 counts and viral load tests were undetectable. During this time, he developed oral candidiasis for which nystatin wash 100,000 units suspension was given to be gargled 3 times a day. After 12 months of MDT, lesions became more edematous and erythematous with severe paresthesia of the bilateral lower extremities (Fig 1, *B-1-B-3*). A repeat biopsy showed more epithelioid histiocytes as compared to previous biopsy done in 2022 (Fig 2), and repeat slit-skin smear showed a bacillary index of 0.5. Due to the undetectable CD4 count, prednisone was gradually tapered and eventually discontinued. MDT was extended for another 3 months, and zinc gluconate supplements were started to support immune function. The lesions remained edematous and erythematous after discontinuation of prednisone. A trial of short-course, high-dose clofazimine (300 mg/day for the first month, followed by 200 mg/day on the second month, and 100 mg/day on the third month) was initiated alongside the standard MDT dose of clofazimine 50 mg/daily.

After 3 months of extended MDT treatment with high-dose clofazimine (April 2024), the lesions flattened, became less erythematous, and turned into brown patches (Fig 1, *C-1-C-3*). MDT was discontinued and there was no recurrence of Type 1 lepra reaction to date. His repeat viral load remained undetectable and his CD4 counts increased to 288 cells/mm^3^. Lesions 1 year post-treatment remained flat, but were less erythematous and less pigmented (Fig 1, *D-1-D-3*).

## Discussion

PLHIV with leprosy have a similar, and overall increased, risk of Type 1 lepra reactions compared to HIV-negative individuals.^7^ These reactions are delayed hypersensitivity reactions that predominantly occur in borderline leprosy, but can also be seen when there is an improvement in the host’s immunity.^1^ Lesions are Th1-mediated and express proinflammatory interferon γ, interleukin-12, and the oxygen-free radical producer inducible nitric oxide synthase.^8^ Type 1 reactions are more frequent than Type 2 reactions in both PLHIV and the general leprosy population.^9^

Clofazimine is a mild anti-inflammatory used in Type 2 lepra reactions, acting by promoting E-series prostaglandins from neutrophils and monocytes.^10^ Its use in the treatment of Type 1 lepra reactions is unclear. It is also one of the medicines in the MDT taken with doses of 300 mg/month and 50 mg/daily with slow bactericidal effect on *Mycobacterium leprae*. It may cause pinkish-brown skin discoloration and severe gastrointestinal symptoms, sometimes requiring dose adjustment. Effects stem from pigment deposition and intestinal crystal formation. No standardized guidelines are available for monitoring and are highly physician-based.^10^

Alternative treatments for Type 1 lepra reactions include cyclosporine, which showed promise, and azathioprine or low-dose methotrexate as possible steroid-sparing agents. Splinting with analgesia may aid in managing severe neuritis.^1^^,^^10^ However, all of these medications have immunosuppressive effects and could potentially lead to further immunosuppression in PLHIV patients.

## Conclusion

Considering the scarcity of literature in the treatment of Type 1 lepra reaction among PLHIV patients with leprosy, and the case presented, short-course, high-dose clofazimine may be considered as a non-immunosuppressive treatment alternative for patients with contraindications to chronic systemic steroid use. However, more studies are needed to validate the efficacy and safety of this regimen.

## Conflicts of interest

None disclosed.
